# Benzotriazolium picrate

**DOI:** 10.1107/S1600536811018307

**Published:** 2011-05-20

**Authors:** Bo Zeng, Ji Li, Guo-dong Wang

**Affiliations:** a Yun Nan Chemical Research Institute, Yun Tian Hua Group, Kunming 650028, People’s Republic of China

## Abstract

In the crystal structure of the title compound, C_6_H_6_N_3_^+^·C_6_H_2_N_3_O_7_^−^, anions and cations are linked into chains along [010] by inter­molecular N—H⋯O hydrogen bonds. These chains are further stabilized by weak C—H⋯O hydrogen bonds and π–π stacking inter­actions with a centroid–centroid distance of 3.908 (1) Å.

## Related literature

For applications of imidazolium-based picrate salts, see: Sikder & Sikder (2004 ▶). For related structures, see: Jin *et al.* (2008 ▶); Hashizume *et al.* (2001 ▶); Li (2007 ▶); Moreno-Fuquen *et al.* (2011 ▶); Pi *et al.* (2009 ▶).
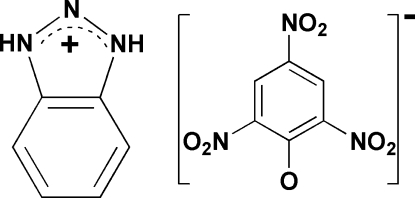


## Experimental

### 

#### Crystal data

C_6_H_6_N_3_^+^·C_6_H_2_N_3_O_7_^−^
*M* *_r_* = 348.24
Monoclinic, 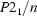

*a* = 14.4113 (15) Å
*b* = 3.7608 (4) Å
*c* = 24.941 (3) Å
β = 90.598 (2)°
*V* = 1351.7 (2) Å^3^
*Z* = 4
Mo *K*α radiation
μ = 0.14 mm^−1^
*T* = 298 K
0.35 × 0.08 × 0.06 mm

#### Data collection

Bruker SMART APEX CCD area-detector diffractometer
Absorption correction: multi-scan (*SADABS*; Sheldrick, 1997 ▶) *T*_min_ = 0.941, *T*_max_ = 0.991
10116 measured reflections
2793 independent reflections
1772 reflections with *I* > 2σ(*I*)
*R*_int_ = 0.068

#### Refinement

*R*[*F* ^2^ > 2σ(*F* ^2^)] = 0.062
*wR*(*F* ^2^) = 0.160
*S* = 1.04
2793 reflections
232 parameters
H atoms treated by a mixture of independent and constrained refinement
Δρ_max_ = 0.26 e Å^−3^
Δρ_min_ = −0.22 e Å^−3^

### 

Data collection: *SMART* (Bruker, 2001 ▶); cell refinement: *SAINT* (Bruker, 2001 ▶); data reduction: *SAINT*; program(s) used to solve structure: *SHELXS97* (Sheldrick, 2008 ▶); program(s) used to refine structure: *SHELXL97* (Sheldrick, 2008 ▶); molecular graphics: *PLATON* (Spek, 2009 ▶); software used to prepare material for publication: *SHELXTL* (Sheldrick, 2008 ▶).

## Supplementary Material

Crystal structure: contains datablocks global, I. DOI: 10.1107/S1600536811018307/lh5251sup1.cif

Structure factors: contains datablocks I. DOI: 10.1107/S1600536811018307/lh5251Isup2.hkl

Supplementary material file. DOI: 10.1107/S1600536811018307/lh5251Isup3.cml

Additional supplementary materials:  crystallographic information; 3D view; checkCIF report

## Figures and Tables

**Table 1:** Hydrogen-bond geometry (Å, °)

*D*—H⋯*A*	*D*—H	H⋯*A*	*D*⋯*A*	*D*—H⋯*A*
N4—H4⋯O1^i^	0.85 (3)	2.04 (3)	2.799 (3)	148 (3)
N4—H4⋯O2^i^	0.85 (3)	2.16 (3)	2.791 (3)	131 (3)
N6—H6⋯O1	0.94 (3)	1.87 (3)	2.770 (3)	160 (3)
N6—H6⋯O6	0.94 (3)	2.48 (3)	3.090 (3)	123 (2)
C8—H8⋯O2^i^	0.93	2.57	3.148 (4)	121
C11—H11⋯O6	0.93	2.56	3.200 (4)	127

Symmetry code: (i) 
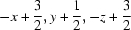
.

**Fig. 1.**
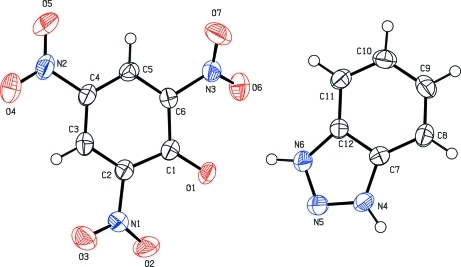
The molecular structure of (I) with displacement ellipsoids drawn at the 50% probability level.

**Fig. 2.**
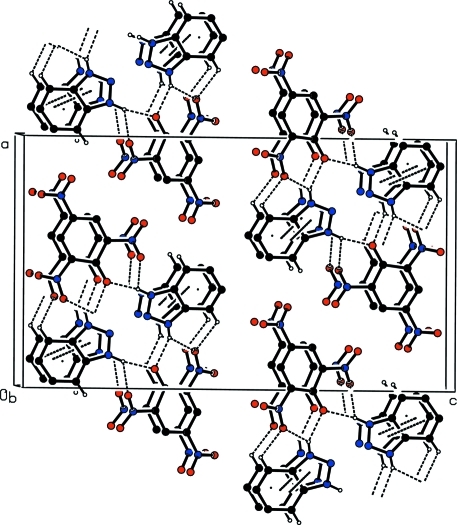
Part of the crystal structure showing the one-dimensional structure formed by a combination of N—H···O and C—H···O hydrogen bonds and π–π interactions (dashed lines). Hydrogen atoms not involved in the motif have been omitted for clarity.

## supplementary crystallographic information

### Comment

Imidazolium-based picrate salts are good candidates for energetic materials (Sikder and Sikder, 2004). Herein we present the crystal structure of the title compound (I) .

The asymmetric unit of the title compound is shown in Fig. 1. During the preparation a phenolic proton was transfered to a triazole nitrogen atom, forming the 1:1 organic salt. In the picrate anion, the bond distances of C1—O1 = 1.251 (3) Å, C1—C2= 1.446 (4)Å and C1—C6 = 1.454 (4)Å may be affected by the withdrawing electron effects of the three nitro groups and are comparable to those in some related structures (Moreno-Fuquen *et al.*, 2011; Pi *et al.*, 2009; Jin *et al.*, 2008; Li, 2007).

In the crystal, the components are linked by a combination of four N—H···O and two C—H···O hydrogen bonds into a one-dimensional structure along [010] which is further stabilized by one π–π interaction between symmetry-related triazole and benzene rings [*Cg*1—*Cg*2^iii^ = 3.908 (1) Å, symmetry code (iii): *x*, 1 + *y*, *z where Cg*1 is the centroid of the triazole ring and *Cg*2 is the centroid defined by atoms C7—C12, respectively] (Fig.2). The crystal structure also contains a short N···N contact ca. 2.89Å. This type of contact is also seen in benzotriazole-3-nitrobenzoic acid (Hashizume *et al.*, 2001).

### Experimental

A mixture of benztriazole (0.238 g, 2 mmol) and picric acid (0.458 g, 2 mmol) was dissolved in 30 ml water at ambient condition. The resultant solution was allowed to stand for two weeks. Yellow needles were obtained at the bottom of the vessel.

### Refinement

All C-bound hydrogen atoms were included in their ideal positions and refined with  C—H= 0.93Å .The U_iso_(H) values were set 1.2U_eq_(C). The nitrogen bonded hydrogen atoms were found in the difference Fourier maps and then refined freely with U_iso_(H)=1.2U_eq_(N).

### Crystal data

**Table:**

C_6_H_6_N_3_^+^·C_6_H_2_N_3_O_7_^−^	*F*(000) = 712
*M**_r_* = 348.24	*D*_x_ = 1.711 Mg m^−^^3^
Monoclinic, *P*2_1_/*n*	Mo *K*α radiation, λ = 0.71073 Å
Hall symbol: -P 2yn	Cell parameters from 1240 reflections
*a* = 14.4113 (15) Å	θ = 2.8–20.3°
*b* = 3.7608 (4) Å	µ = 0.14 mm^−^^1^
*c* = 24.941 (3) Å	*T* = 298 K
β = 90.598 (2)°	Needle, yellow
*V* = 1351.7 (2) Å^3^	0.35 × 0.08 × 0.06 mm
*Z* = 4	

### Data collection

**Table:**

Bruker SMART APEX CCD area-detector diffractometer	2793 independent reflections
Radiation source: fine focus sealed Siemens Mo tube	1772 reflections with *I* > 2σ(*I*)
graphite	*R*_int_ = 0.068
0.3° wide ω scans	θ_max_ = 26.5°, θ_min_ = 1.6°
Absorption correction: multi-scan (*SADABS*; Sheldrick, 1997)	*h* = −18→18
*T*_min_ = 0.941, *T*_max_ = 0.991	*k* = −4→4
10116 measured reflections	*l* = −28→31

### Refinement

**Table:**

Refinement on *F*^2^	Primary atom site location: structure-invariant direct methods
Least-squares matrix: full	Secondary atom site location: difference Fourier map
*R*[*F*^2^ > 2σ(*F*^2^)] = 0.062	Hydrogen site location: inferred from neighbouring sites
*wR*(*F*^2^) = 0.160	H atoms treated by a mixture of independent and constrained refinement
*S* = 1.04	*w* = 1/[σ^2^(*F*_o_^2^) + (0.075*P*)^2^] where *P* = (*F*_o_^2^ + 2*F*_c_^2^)/3
2793 reflections	(Δ/σ)_max_ < 0.001
232 parameters	Δρ_max_ = 0.26 e Å^−^^3^
0 restraints	Δρ_min_ = −0.22 e Å^−^^3^

### Special details

**Table:**

Geometry. All e.s.d.'s (except the e.s.d. in the dihedral angle between two l.s. planes) are estimated using the full covariance matrix. The cell e.s.d.'s are taken into account individually in the estimation of e.s.d.'s in distances, angles and torsion angles; correlations between e.s.d.'s in cell parameters are only used when they are defined by crystal symmetry. An approximate (isotropic) treatment of cell e.s.d.'s is used for estimating e.s.d.'s involving l.s. planes.
Refinement. Refinement of *F*^2^ against ALL reflections. The weighted *R*-factor *wR* and goodness of fit *S* are based on *F*^2^, conventional *R*-factors *R* are based on *F*, with *F* set to zero for negative *F*^2^. The threshold expression of *F*^2^ > σ(*F*^2^) is used only for calculating *R*-factors(gt) *etc*. and is not relevant to the choice of reflections for refinement. *R*-factors based on *F*^2^ are statistically about twice as large as those based on *F*, and *R*- factors based on ALL data will be even larger.

### Fractional atomic coordinates and isotropic or equivalent isotropic displacement parameters (Å^2^)

**Table:**

	*x*	*y*	*z*	*U*_iso_*/*U*_eq_	
C1	0.99963 (18)	0.3370 (8)	0.66441 (12)	0.0292 (7)	
C2	1.01146 (18)	0.4733 (8)	0.61071 (11)	0.0306 (7)	
C3	1.09520 (19)	0.5092 (8)	0.58563 (12)	0.0339 (7)	
H3	1.0979	0.6016	0.5511	0.041*	
C4	1.17487 (18)	0.4073 (8)	0.61202 (12)	0.0329 (7)	
C5	1.17278 (19)	0.2734 (8)	0.66338 (11)	0.0318 (7)	
H5	1.2273	0.2056	0.6809	0.038*	
C6	1.08877 (19)	0.2420 (8)	0.68823 (11)	0.0298 (7)	
N1	0.93044 (16)	0.5892 (7)	0.57961 (10)	0.0359 (6)	
N2	1.26389 (18)	0.4342 (8)	0.58494 (12)	0.0447 (7)	
N3	1.09156 (17)	0.1027 (7)	0.74265 (10)	0.0365 (6)	
C7	0.82043 (17)	0.5020 (7)	0.87509 (12)	0.0282 (7)	
C8	0.8186 (2)	0.4915 (8)	0.93112 (12)	0.0377 (8)	
H8	0.7672	0.5650	0.9504	0.045*	
C9	0.8973 (2)	0.3662 (9)	0.95568 (12)	0.0412 (8)	
H9	0.8997	0.3555	0.9929	0.049*	
C10	0.9752 (2)	0.2525 (9)	0.92626 (13)	0.0408 (8)	
H10	1.0272	0.1713	0.9449	0.049*	
C11	0.97677 (19)	0.2573 (8)	0.87192 (12)	0.0365 (8)	
H11	1.0281	0.1809	0.8528	0.044*	
C12	0.89671 (18)	0.3838 (8)	0.84644 (11)	0.0291 (7)	
N4	0.75692 (16)	0.6019 (7)	0.83701 (10)	0.0347 (6)	
H4	0.702 (2)	0.678 (9)	0.8423 (12)	0.042*	
N5	0.78784 (16)	0.5608 (7)	0.78832 (10)	0.0388 (7)	
N6	0.87157 (16)	0.4287 (7)	0.79375 (10)	0.0354 (6)	
H6	0.896 (2)	0.343 (8)	0.7615 (13)	0.043*	
O1	0.92425 (12)	0.3151 (6)	0.68863 (8)	0.0385 (6)	
O2	0.85380 (14)	0.4837 (7)	0.59216 (10)	0.0604 (8)	
O3	0.94256 (15)	0.7832 (7)	0.54123 (10)	0.0577 (7)	
O4	1.26640 (16)	0.6034 (8)	0.54315 (11)	0.0691 (8)	
O5	1.33151 (15)	0.2909 (8)	0.60522 (10)	0.0625 (8)	
O6	1.02722 (16)	−0.0819 (6)	0.75823 (9)	0.0497 (6)	
O7	1.15869 (17)	0.1753 (8)	0.77088 (10)	0.0642 (8)	

### Atomic displacement parameters (Å^2^)

**Table:**

	*U*^11^	*U*^22^	*U*^33^	*U*^12^	*U*^13^	*U*^23^
C1	0.0274 (14)	0.0287 (17)	0.0316 (17)	−0.0046 (12)	0.0038 (12)	−0.0064 (13)
C2	0.0299 (15)	0.0319 (17)	0.0301 (17)	−0.0041 (12)	0.0013 (12)	−0.0065 (13)
C3	0.0373 (16)	0.0361 (18)	0.0284 (17)	−0.0073 (14)	0.0062 (13)	−0.0048 (14)
C4	0.0278 (15)	0.0390 (18)	0.0320 (17)	−0.0041 (13)	0.0066 (12)	−0.0071 (14)
C5	0.0276 (15)	0.0345 (17)	0.0332 (18)	0.0027 (12)	−0.0003 (13)	−0.0070 (14)
C6	0.0340 (15)	0.0298 (17)	0.0255 (16)	−0.0003 (13)	0.0021 (13)	−0.0001 (13)
N1	0.0351 (14)	0.0390 (16)	0.0337 (16)	0.0006 (12)	−0.0004 (12)	0.0026 (13)
N2	0.0349 (15)	0.060 (2)	0.0397 (17)	−0.0089 (14)	0.0123 (13)	−0.0074 (15)
N3	0.0382 (14)	0.0397 (16)	0.0317 (15)	0.0063 (12)	0.0051 (12)	0.0005 (12)
C7	0.0265 (14)	0.0245 (16)	0.0337 (17)	−0.0041 (12)	−0.0012 (12)	−0.0025 (13)
C8	0.0354 (16)	0.044 (2)	0.0342 (19)	−0.0017 (14)	0.0082 (14)	−0.0057 (15)
C9	0.0470 (19)	0.049 (2)	0.0280 (18)	−0.0047 (16)	−0.0039 (14)	0.0009 (15)
C10	0.0352 (16)	0.047 (2)	0.040 (2)	0.0036 (14)	−0.0094 (14)	0.0000 (16)
C11	0.0284 (15)	0.0410 (19)	0.040 (2)	0.0030 (13)	0.0030 (13)	−0.0049 (15)
C12	0.0281 (14)	0.0314 (17)	0.0277 (17)	−0.0056 (12)	0.0006 (12)	−0.0029 (13)
N4	0.0254 (12)	0.0407 (16)	0.0381 (16)	0.0031 (11)	0.0025 (12)	−0.0029 (12)
N5	0.0349 (14)	0.0464 (17)	0.0349 (16)	0.0059 (12)	−0.0020 (12)	−0.0040 (13)
N6	0.0336 (14)	0.0434 (16)	0.0294 (15)	0.0026 (12)	0.0024 (11)	−0.0062 (12)
O1	0.0259 (11)	0.0570 (15)	0.0328 (12)	−0.0052 (9)	0.0082 (9)	−0.0033 (10)
O2	0.0279 (12)	0.086 (2)	0.0668 (17)	−0.0040 (12)	0.0000 (11)	0.0266 (15)
O3	0.0533 (15)	0.0726 (19)	0.0471 (16)	−0.0052 (13)	−0.0047 (12)	0.0238 (14)
O4	0.0527 (15)	0.100 (2)	0.0548 (17)	−0.0080 (14)	0.0227 (13)	0.0202 (16)
O5	0.0281 (12)	0.093 (2)	0.0662 (18)	0.0041 (12)	0.0069 (12)	−0.0023 (15)
O6	0.0542 (14)	0.0533 (16)	0.0418 (14)	−0.0085 (12)	0.0131 (11)	0.0082 (12)
O7	0.0510 (15)	0.096 (2)	0.0457 (16)	−0.0068 (14)	−0.0160 (12)	0.0166 (14)

### Geometric parameters (Å, °)

**Table:**

C1—O1	1.251 (3)	N3—O6	1.225 (3)
C1—C2	1.446 (4)	C7—N4	1.365 (4)
C1—C6	1.454 (4)	C7—C12	1.390 (4)
C2—C3	1.372 (4)	C7—C8	1.398 (4)
C2—N1	1.461 (4)	C8—C9	1.366 (4)
C3—C4	1.372 (4)	C8—H8	0.9300
C3—H3	0.9300	C9—C10	1.413 (4)
C4—C5	1.377 (4)	C9—H9	0.9300
C4—N2	1.459 (3)	C10—C11	1.356 (4)
C5—C6	1.371 (4)	C10—H10	0.9300
C5—H5	0.9300	C11—C12	1.395 (4)
C6—N3	1.455 (4)	C11—H11	0.9300
N1—O2	1.218 (3)	C12—N6	1.370 (4)
N1—O3	1.218 (3)	N4—N5	1.307 (3)
N2—O5	1.219 (3)	N4—H4	0.85 (3)
N2—O4	1.222 (3)	N5—N6	1.311 (3)
N3—O7	1.222 (3)	N6—H6	0.94 (3)
			
O1—C1—C2	125.6 (3)	N4—C7—C8	133.2 (3)
O1—C1—C6	123.7 (3)	C12—C7—C8	121.8 (3)
C2—C1—C6	110.7 (2)	C9—C8—C7	115.7 (3)
C3—C2—C1	124.8 (3)	C9—C8—H8	122.1
C3—C2—N1	115.5 (3)	C7—C8—H8	122.1
C1—C2—N1	119.7 (2)	C8—C9—C10	122.1 (3)
C2—C3—C4	119.3 (3)	C8—C9—H9	119.0
C2—C3—H3	120.3	C10—C9—H9	119.0
C4—C3—H3	120.3	C11—C10—C9	122.5 (3)
C3—C4—C5	121.5 (3)	C11—C10—H10	118.8
C3—C4—N2	119.6 (3)	C9—C10—H10	118.8
C5—C4—N2	118.9 (3)	C10—C11—C12	115.9 (3)
C6—C5—C4	118.7 (3)	C10—C11—H11	122.0
C6—C5—H5	120.7	C12—C11—H11	122.0
C4—C5—H5	120.7	N6—C12—C7	104.5 (2)
C5—C6—C1	125.1 (3)	N6—C12—C11	133.5 (3)
C5—C6—N3	115.9 (2)	C7—C12—C11	122.0 (3)
C1—C6—N3	119.0 (2)	N5—N4—C7	112.4 (2)
O2—N1—O3	122.4 (3)	N5—N4—H4	121 (2)
O2—N1—C2	119.2 (3)	C7—N4—H4	127 (2)
O3—N1—C2	118.4 (2)	N4—N5—N6	105.7 (2)
O5—N2—O4	123.6 (3)	N5—N6—C12	112.3 (2)
O5—N2—C4	118.7 (3)	N5—N6—O1	102.81 (18)
O4—N2—C4	117.7 (3)	C12—N6—O1	144.73 (19)
O7—N3—O6	122.8 (3)	N5—N6—H6	112.9 (19)
O7—N3—C6	118.1 (3)	C12—N6—H6	133.3 (19)
O6—N3—C6	119.1 (2)	C1—O1—N6	134.01 (19)
N4—C7—C12	105.0 (2)		
			
O1—C1—C2—C3	177.1 (3)	C5—C6—N3—O6	−146.4 (3)
C6—C1—C2—C3	−0.1 (4)	C1—C6—N3—O6	33.7 (4)
O1—C1—C2—N1	−2.0 (4)	N4—C7—C8—C9	178.9 (3)
C6—C1—C2—N1	−179.3 (3)	C12—C7—C8—C9	1.5 (4)
C1—C2—C3—C4	0.7 (5)	C7—C8—C9—C10	−0.4 (5)
N1—C2—C3—C4	179.9 (3)	C8—C9—C10—C11	−0.5 (5)
C2—C3—C4—C5	−0.7 (5)	C9—C10—C11—C12	0.3 (5)
C2—C3—C4—N2	178.1 (3)	N4—C7—C12—N6	0.7 (3)
C3—C4—C5—C6	0.1 (4)	C8—C7—C12—N6	178.7 (3)
N2—C4—C5—C6	−178.7 (3)	N4—C7—C12—C11	−179.8 (3)
C4—C5—C6—C1	0.6 (4)	C8—C7—C12—C11	−1.8 (4)
C4—C5—C6—N3	−179.3 (3)	C10—C11—C12—N6	−179.8 (3)
O1—C1—C6—C5	−177.8 (3)	C10—C11—C12—C7	0.8 (4)
C2—C1—C6—C5	−0.5 (4)	C12—C7—N4—N5	−1.0 (3)
O1—C1—C6—N3	2.0 (4)	C8—C7—N4—N5	−178.6 (3)
C2—C1—C6—N3	179.3 (3)	C7—N4—N5—N6	0.9 (3)
C3—C2—N1—O2	160.8 (3)	N4—N5—N6—C12	−0.4 (3)
C1—C2—N1—O2	−20.0 (4)	N4—N5—N6—O1	176.53 (19)
C3—C2—N1—O3	−17.7 (4)	C7—C12—N6—N5	−0.2 (3)
C1—C2—N1—O3	161.4 (3)	C11—C12—N6—N5	−179.6 (3)
C3—C4—N2—O5	−168.3 (3)	C7—C12—N6—O1	−175.0 (3)
C5—C4—N2—O5	10.5 (4)	C11—C12—N6—O1	5.6 (6)
C3—C4—N2—O4	12.4 (4)	C2—C1—O1—N6	−140.9 (3)
C5—C4—N2—O4	−168.8 (3)	C6—C1—O1—N6	36.0 (4)
C5—C6—N3—O7	33.4 (4)	N5—N6—O1—C1	144.9 (3)
C1—C6—N3—O7	−146.5 (3)	C12—N6—O1—C1	−40.0 (5)

### Hydrogen-bond geometry (Å, °)

**Table:**

*D*—H···*A*	*D*—H	H···*A*	*D*···*A*	*D*—H···*A*
N4—H4···O1^i^	0.85 (3)	2.04 (3)	2.799 (3)	148 (3)
N4—H4···O2^i^	0.85 (3)	2.16 (3)	2.791 (3)	131 (3)
N6—H6···O1	0.94 (3)	1.87 (3)	2.770 (3)	160 (3)
N6—H6···O6	0.94 (3)	2.48 (3)	3.090 (3)	123 (2)
C8—H8···O2^i^	0.93	2.57	3.148 (4)	121
C11—H11···O6	0.93	2.56	3.200 (4)	127

Symmetry codes: (i) −*x*+3/2, *y*+1/2, −*z*+3/2.
